# Fractional Anisotropy of Cingulum Cingulate Mediates the Relationship Between Happiness and Work Performance in Healthy Individuals

**DOI:** 10.1002/brb3.70334

**Published:** 2025-02-17

**Authors:** Keisuke Kokubun, Kiyotaka Nemoto, Yoshinori Yamakawa

**Affiliations:** ^1^ Open Innovation Institute Kyoto University Kyoto Japan; ^2^ Graduate School of Management Kyoto University Kyoto Japan; ^3^ Department of Psychiatry, Institute of Medicine University of Tsukuba Tsukuba Japan; ^4^ Institute of Innovative Research Tokyo Institute of Technology Meguro Tokyo Japan; ^5^ ImPACT Program of Council for Science Technology and Innovation (Cabinet Office, Government of Japan) Chiyoda Tokyo Japan; ^6^ Office for Academic and Industrial Innovation Kobe University Kobe Japan; ^7^ Brain Impact Kyoto Japan

**Keywords:** cingulum cingulate, fractional anisotropy, happiness, performance, superior longitudinal fasciculus, work role

## Abstract

**Introduction:**

As competition among companies around the world intensifies, the nature of work and the performance required are becoming more complex. In parallel with this, there is growing attention on happiness and well‐being as factors related to improving employee performance. However, little is known about the relationship between happiness and the brain and work performance in healthy people.

**Methods:**

Therefore, we analyzed the correlations between the nine categories of work role performance (WRP), the subjective happiness scale (SHS), and four regions of fractional anisotropy (FA), an index reflecting brain microstructure that has been shown to be related to apathy in previous studies.

**Results:**

It was shown that the cingulum cingulate (CCI) and the superior longitudinal fasciculus (SLF) correlated with the WRP and its facets in a manner consistent with their respective functions. In particular, the CCI was found to be extensively correlated with the facets of the WRP and to have a partially mediating effect on the relationship between the SHS and the WRP.

**Conclusion:**

This study is the first to show that indicators reflecting healthy individuals’ happiness and brain microstructure, which are related to a variety of nonwork factors, are positively correlated with the diverse roles and performance that characterize modern work.

## Graphical abstract

It was shown that cingulum cingulate (CCI) partially mediates the relationship between subjective happiness scale (SHS) and work role performance (WRP).

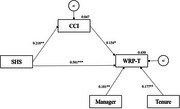

## 1 Limitation

Because this study was conducted using a cross‐sectional analysis, the causal relationship of the variables is unclear. In addition, because the study was conducted on Japanese subjects, caution should be exercised when applying the results to people in other countries. Furthermore, the small sample size reduces the generalizability of the results. Future research should verify the results of this study through cross‐sectional and longitudinal studies that include a large number of participants of diverse nationalities.

## 2 Introduction

As globalization intensifies competition between companies, the content of work and the performance required are becoming more complex. For this reason, although many jobs were once evaluated in terms of proficiency, as the content of work changes, the importance of adaptability to change and the proactiveness to bring about change is increasing. In parallel, the importance of work done in teams and organizations is increasing from work that can be done individually (Griffin, Neal, and Parker 2007; Neal et al. 2012). Therefore, Griffin, Neal, and Parker (2007) proposed work role performance (WRP), which is a set of nine aspects of performance required according to roles, by cross‐classifying performance (proficient, adaptive, and proactive) and role (individual, team, and organizational) yielding: individual task proficiency (WRP‐1), team member proficiency (WRP‐2), organization member proficiency (WRP‐3), individual task adaptivity (WRP‐4), team member adaptivity (WRP‐5), organization member adaptivity (WRP‐6), individual task proactivity (WRP‐7), team member proactivity (WRP‐8), and organization member proactivity (WRP‐9). Here, “proficient” refers to the degree to which one meets the requirements of one's role. Meanwhile, “adaptive” refers to the degree to which one adapts to changes in the work system or role, and “proactive” refers to the degree to which one takes autonomous action to predict or initiate changes in the work system or role.

These performances are different depending on the individual, team, and organizational roles. For example, individual proficiency includes meeting the requirements of the individual employee's role, whereas team and organizational proficiency includes meeting the requirements of the role as a team or organizational member. Team requirements include supporting other team members and coordinating activities. Meanwhile, organizational requirements include maintaining or improving the image of the organization as held by stakeholders. Similarly, individual adaptability includes adapting to changes that affect one's role as an individual, whereas team and organizational adaptability includes adapting to changes that affect one's role as a team or organizational member. Individual proactiveness includes initiating changes within one's role, whereas team and organizational proactiveness includes initiating changes in the team or organization (Griffin, Neal, and Parker 2007).

The developers of the WRP showed that openness to change is positively correlated with the adaptability of individuals, teams, and organizational members, and that organizational commitment is positively correlated with all facets of the WRP (Griffin, Neal, and Parker 2007). Recent studies have also shown that work engagement (Dhir and Shukla 2019; Fletcher 2016) and personal role engagement (Fletcher 2016) are positively correlated with individual, team, and organizational performance of the WRP. Meanwhile, Neal et al. (2012) attempted to clarify the relationship between the Big Five model of personality traits and the WRP and showed that openness to experience is positively correlated with individual and organizational proactiveness.

In parallel with changes in work style, happiness has been attracting attention from academia and industry in recent years as a factor related to work performance. Previous studies have shown that happiness has a positive effect on employees’ mental health, enhancing their creativity, imagination, and problem‐solving abilities, and improving their performance (Al‐Hawari, Bani‐Melhem, and Shamsudin 2019; Bani‐Melhem, Zeffane, and Albaity 2018; Pannells and Claxton 2008; Semedo, Coelho, and Ribeiro 2017; Steptoe 2019). Recent studies have also shown that happiness, measured by the subjective happiness scale (SHS), a scale developed by Lyubomirsky and Lepper (1999) and used in many studies, is positively correlated with employees’ engagement in the creative process and performance (Khan and Abbas 2022). In addition, there has been a certain accumulation of research on the relationship among happiness, well‐being, life satisfaction, and the brain structure of healthy individuals, showing that cingulate cortex (Matsunaga et al. 2016) or fractional anisotropy (FA) in related brain regions (Kokubun, Nemoto, and Yamakawa 2022; Maeda et al. 2021) are associated with these positive indicators.

Research is also underway into the relationship between apathy, which manifests as a lack of emotion, interest, and motivation and is seen in many patients with neurodegenerative and psychiatric disorders such as Alzheimer's disease (AD), and brain structures. Apathy is defined as a quantitative reduction in spontaneous or goal‐directed behavior and occurs when brain circuits in the frontal cortex and basal ganglia are affected (Levy 2012). Apathy is frequently accompanied by “cognitive inertia,” which refers to difficulties in retrieving words, retrieving information from declarative memory, generating new rules and strategies, and shifting from one mental and behavioral set to another (Levy 2012). Therefore, apathy can be predicted to make it difficult for workers to meet the criteria of the WRP, that is, to be proficient, adaptive, and proactive. Previous studies in AD patients have shown a relationship between apathy and the frontal cortex (Bruen et al. 2008; Craig et al. 1996), anterior cingulate cortex (ACC) (Bruen et al. 2008; Migneco et al. 2001), thalamus (Marshall et al. 2007), and so forth. In addition, because AD is associated with white matter (WM) abnormalities (Agosta et al. 2011), there has been an increase in diffusion tensor imaging (DTI) studies (Powers et al. 2014). Studies of AD patients have shown an association between the severity of apathy and FA in the uncinate fasciculus (UFA), corpus callosum (CCA), superior longitudinal fasciculus (SLF), and cingulum cingulate (CCI) (Hahn et al. 2013; Kim et al. 2011; Smith et al. 2006; Starkstein et al. 1993). In addition, recent studies in healthy individuals have shown that lifestyle factors such as diet and exercise (Kokubun, Nemoto, and Yamakawa 2024), sleep quality (Jamieson et al. 2021; Xu et al. 2023), and living environment (Pineda et al. 2021) are associated with FA, suggesting that changes in brain microstructure can occur in individuals before they develop a specific disease due to environmental changes.

In this study, we analyze the relationship between FA and WRP in four regions (UFA, CCA, SLF, and CCI) that have been shown to be related to apathy in healthy subjects. Furthermore, we test whether FA partially mediates the relationship between happiness measured by the SHS and WRP.

## 3 Materials and Methods

### 3.1 Participants

A total of 137 participants (106 men, 31 women) aged 22–63 years (mean = 42.46, standard deviation (SD) = 10.856) were recruited to the Tokyo Institute of Technology from September to October 2019. Respondents were randomly recruited from the public by private recruitment agencies and the Brain Healthcare Quotient (BHQ) Consortium, a study group aiming to utilize brain information in industry (BHQ Inc. 2024). According to the self‐report, no subjects recruited had records of neurological, psychiatric, or other medical conditions that could affect the central nervous system. Brain images were taken using magnetic resonance imaging (MRI), and the participants then answered a questionnaire. All methods were carried out according to the relevant guidelines, regulations, and principles of the Declaration of Helsinki.

### 3.2 MRI Data Acquisition

All MRI data were obtained using a 3‐T Siemens scanner (MAGNETOM Prisma, Siemens, Munich, Germany), which has a 32‐channel head array coil and a three‐dimensional (3D) T1‐weighted magnetization‐prepared and rapid‐acquisition gradient echo pulse sequence. The parameters include repetition time (TR), 1900 ms; echo time (TE), 2.52 ms; inversion time (TI), 900 ms; flip angle, 9°; matrix size, 256 × 256; field of view (FOV), 256 mm; and slice thickness, 1 mm. DTI data were collected by spin‐echo echo‐planar imaging (SE‐EPI) using generalized auto‐calibrating partially parallel acquisitions (GRAPPA). The image slices were parallel to the orbitomeatal (OM) line with the parameters: TR = 14,100 ms; TE = 81 ms; flip angle = 90°; matrix size = 114 × 114; FOV = 224 mm; slice thickness = 2 mm. The baseline image (*b* = 0 s/mm^2^) and 30 different diffusion directions were obtained with a *b* value of 1000 s/mm^2^.

### 3.3 MRI Data Analysis

DTI data were preprocessed using the FMRIB software library (FSL) 5.0.11 (Jenkinson et al. 2012). First, we aligned all the diffuse images and used eddy corrections. Following these modifications, the FA images were calculated using a DTIFit. After FA images were spatially normalized into the standard Montreal Neurological Laboratory (MNI) space using FLIRT and FNIRT, individual FA quotient images were generated using the formula, 100 + 15 × (individual FA − mean)/SD, to create a participant‐specific FA brain healthcare quotient (FA‐BHQ) using the Johns Hopkins University (JHU) DTI‐based WM atlas (Mori et al. 2008). See Nemoto et al. (2017) for more information. Previous studies have shown that FA‐BHQ is positively correlated with cognitive function (Kokubun, Nemoto, and Yamakawa 2020), happiness (Kokubun, Nemoto, and Yamakawa 2022), and negatively with anxiety (Pineda et al. 2021) and fatigue (Yan et al. 2024). Additionally, longitudinal studies have shown that participation in human resource development training (Kokubun et al. 2020) and the use of smartphone apps for health promotion (Kokubun, Nemoto, and Yamakawa 2024) increase FA‐BHQ. In this study, we investigated the regional FA‐BHQ in UFA, CCA, SLF, and CCI from the perspective of ROI.

In this study, in addition to FA‐BHQ, we use the gray‐matter brain healthcare quotient (GM‐BHQ), which reflects the gray matter volume (GMV) of the whole brain, as a covariate. We use this covariate because FA is thought to decrease mainly due to brain atrophy and the formation of lesions (Vernooij et al. 2008). In other words, by removing the influence of GMV (Dorszewska 2013), which is thought to atrophy due to aging‐related neuronal aging and reduced plasticity, FA can be used in the analysis model as an index that is more sensitive to the influence of lesions. Such measures can be justified, for example, by previous studies that claim that reduced WM integrity in CCI and SLF is one of the characteristics of the brains of autistic patients (Ameis and Catani 2015; Travers et al. 2012). For the calculation method of GM‐BHQ, please refer to Kokubun, Yamakawa, and Nemoto (2023).

### 3.4 Psychology Test

#### 3.4.1 Work Role Performance

We used the Japanese version of the WRP (Ota et al. 2016), developed by Griffin, Neal, and Parker (2007). This scale combines three roles (individual, team, and organization) and three performances (proficient, adaptive, and proactive), with a total of nine subdimensions, WRP‐1 to WRP‐9. Each dimension consists of three items, making a total of 27 items. Examples include: “ensured tasks were completed properly” (individual task proficiency), “responded constructively to changes in the way his/her team works” (team adaptivity), and “came up with ways of increasing efficiency within the organization” (organizational proactivity). Responses were made on a seven‐point Likert scale ranging from 1 (“very little”) to 7 (a “great deal”). Alpha reliability was 0.703 (WRP‐1), 0.823 (WRP‐2), 0.817 (WRP‐3), 0.730 (WRP‐4), 0.829 (WRP‐5), 0.803 (WRP‐6), 0.899 (WRP‐7), 0.932 (WRP‐8), and 0.961 (WRP‐9), which were sufficiently high. The arithmetic means of the three items and nine subdimentions were used in the analysis as WRPs and WRP‐T, respectively.

#### 3.4.2 Happiness

We use the Japanese version of the SHS (Shimai et al. 2004) developed by Lyubomirsky and Lepper (1999). The SHS was developed to assess subjective happiness and consists of four items rated on a 7‐point Likert scale. The first two items require respondents to rate their general happiness (1 = not a very happy person to 7 = a very happy person) and their happiness compared to their peers (1 = less happy to 7 = more happy). The other two items ask respondents to indicate to what extent they agree with brief descriptions of generally happy and unhappy people (1 = not at all to 7 = a great deal). The variable used in the analysis was the arithmetic mean of the four items. Alpha reliabilities were 0.865, which was sufficiently high.

### 3.5 Data Analysis

First, we estimated correlation coefficients and partial correlation coefficients controlling for sex, age, BMI, GMV, income, education, tenure, and manager for the relationship between the subscales and total scale of WRP, FA in the four regions, and SHS. Next, we tested the mediation effect of FA in the regions with a high correlation with WRP on the relationship between SHS and WRP using path analysis. The criterion for significance was set at 5% for a two‐sided test. All statistical analyses have been performed using IBM SPSS Statistics/AMOS Version 26 (IBM Corp., Armonk, NY, USA).

## 4 Results

**FIGURE 1:**
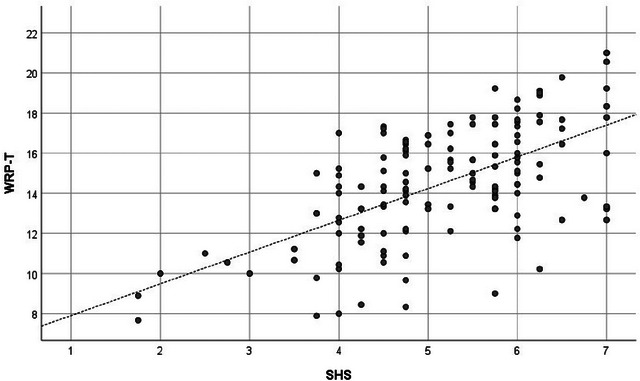
Scatter plot of SHS and WRP‐T. SHS = subjective happiness scale, WRP‐T = total score of work role performance.

**FIGURE 2:**
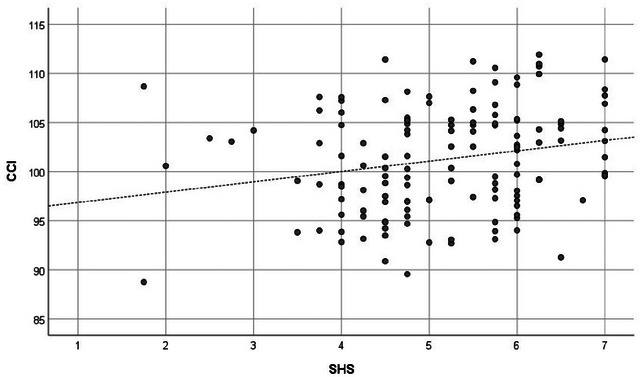
Scatter plot of SHS and CCI. CCI = cingulum cingulate, SHS = subjective happiness scale.

**FIGURE 3:**
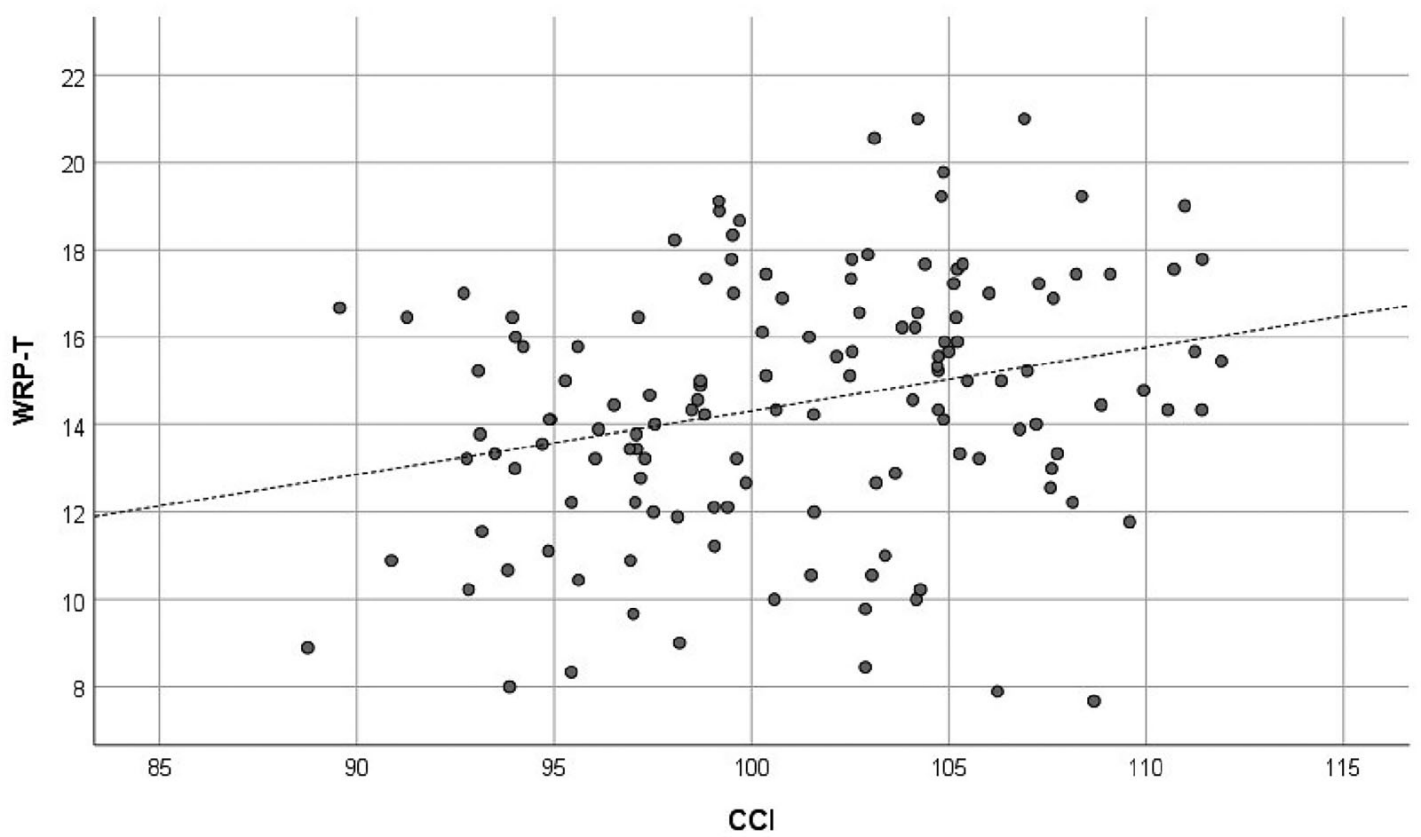
Scatter plot of CCI and WRP‐T. CCI = cingulum cingulate, WRP‐T = total score of work role performance.

Before proceeding with the main analysis, we used Harman's single factor analysis to check whether the variance in the SHS 4 items and WRP 27 items could be mainly attributed to a single factor. The analysis showed that only 45.3% of the variance could be explained by a single factor, which was <50%. Thus, it was established that the data were not affected by common method variance (Podsakoff et al. 2003). Table 1 shows the results. Columns 1 and 2 show the mean and SD, and Columns 3–8 show the usual correlation coefficients. The CCI had significant positive correlations with the SHS (*r* = 0.218; *p* = 0.011), WRP‐T (*r* = 0.268; *p* = 0.002), and seven subscales except for WRP‐2 and WRP‐9. The SLF also had significant positive correlations with the SHS (*r* = 0.248; *p* = 0.003), WRP‐4 (*r* = 0.197; *p* = 0.021), and WRP‐5 (*r* = 0.174; *p* = 0.042). Columns 9–14 show the partial correlation coefficients. Again, the CCI was significantly positively correlated with the SHS (*r* = 0.233; *p* = 0.008), WRP‐T (*r* = 0.266; *p* = 0.002), and seven subscales except for WRP‐2 and WRP‐9. On the other hand, the SLF was significantly positively correlated with the SHS (*r* = 0.264; *p* = 0.002), WRP‐5 (*r* = 0.184; *p* = 0.037), and WRP‐6 (*r* = 0.191; *p* = 0.030). In addition, the SHS had a broad and significant positive correlation with the WRP, regardless of the presence or absence of control variables. Figures 1, 2, 3 show the scatter plots of the relationship between the SHS and the WRP‐T, the SHS and the CCI, and the CCI and the WRP‐T, respectively.

**TABLE 1:** Correlation analysis results.

	1	2	3	4	5	6	7	8	9	10	11	12	13	14
	Mean	SD	Ordinary correlation coefficient						Partial correlation coefficient					
			WRP‐T	SHS	CCI	CCA	SLF	UFA	WRP‐T	SHS	CCI	CCA	SLF	UFA
WRP‐1	13.120	3.593	0.545^***^	0.342^***^	0.187^*^	0.033	0.14	0.077	0.572^***^	0.366^***^	0.178^*^	0.069	0.169	0.072
WRP‐2	16.160	3.270	0.639^***^	0.365^***^	0.140	0.067	0.164	0.055	0.678^***^	0.378^***^	0.135	0.021	0.146	0.065
WRP‐3	14.800	3.477	0.742^***^	0.448^***^	0.256^**^	−0.016	0.085	0.087	0.725^***^	0.436^***^	0.270^**^	0.024	0.127	0.084
WRP‐4	15.410	3.074	0.804^***^	0.524^***^	0.249^**^	0.132	0.197*	−0.014	0.829^***^	0.521^***^	0.265^**^	0.087	0.164	−0.04
WRP‐5	15.100	3.608	0.861^***^	0.590^***^	0.213*	0.062	0.174*	−0.004	0.870^***^	0.579^***^	0.227*	0.039	0.184*	−0.005
WRP‐6	14.260	3.869	0.833^***^	0.586^***^	0.256^**^	−0.002	0.122	−0.010	0.814^***^	0.597^***^	0.259^**^	0.049	0.191*	−0.030
WRP‐7	14.930	3.669	0.815^***^	0.483^***^	0.199*	−0.113	0.039	−0.064	0.801^***^	0.491^***^	0.185*	−0.053	0.072	−0.104
WRP‐8	13.810	4.343	0.839^***^	0.479^***^	0.242^**^	−0.037	0.016	0.062	0.825^***^	0.505^***^	0.225*	0.039	0.074	0.031
WRP‐9	12.820	4.937	0.823^***^	0.403^***^	0.137	−0.112	−0.032	0.039	0.814^***^	0.435^***^	0.124	−0.008	0.038	0.013
WRP‐T	14.488	2.902		0.606^***^	0.268^**^	−0.009	0.118	0.034		0.620^***^	0.266^**^	0.036	0.162	0.013
SHS	5.161	1.112	0.606^***^		0.218*	0.102	0.248^**^	0.048	0.620^***^		0.233^**^	0.084	0.264^**^	0.071
CCI	101.230	5.373	0.268^**^	0.218*		0.518^***^	0.445^***^	0.372^***^	0.266^**^	0.233^**^		0.630^***^	0.512^***^	0.376^***^
CCA	101.705	4.405	−0.009	0.102	0.518^***^		0.627^***^	0.443^***^	0.036	0.084	0.630^***^		0.623^***^	0.518^***^
SLF	101.057	4.529	0.118	0.248^**^	0.445^***^	0.627^***^		0.324^***^	0.162	0.264^**^	0.512^***^	0.623^***^		0.343^***^
UFA	99.662	6.152	0.034	0.048	0.372^***^	0.443^***^	0.324^***^		0.013	0.071	0.376^***^	0.518^***^	0.343^***^	
Sex	1.230	0.420	−0.167	−0.027	−0.110	0.162	0.132	−0.119						
Age	42.460	10.856	0.149	−0.050	0.039	−0.343^***^	−0.273^**^	0.007						
BMI	22.657	3.130	0.106	0.004	−0.028	−0.039	0.035	0.024						
GMV	102.294	7.293	−0.134	0.031	−0.117	0.389^***^	0.165	−0.042						
Income	10.540	3.005	0.209*	0.225^**^	0.020	−0.157	−0.147	−0.043						
Education	16.710	2.246	0.019	0.039	0.078	−0.023	−0.060	0.004						
Tenure	2.990	1.616	.195*	0.056	−0.021	−0.173*	0.008	0.095						
Manager	0.220	0.415	0.229^**^	0.079	0.106	−0.036	−0.100	−0.014						

*Note*: *n* = 137.The annual income was created by allocating 0–15 points for each of the following 16 options: “No income,” “less than 500,000 yen,” “500,000–990,000 yen,” “1–1.49 million yen,” “1.5–1.99 million yen,” “2–2.49 million yen,” “2.5–2.99 million yen,” “3–3.99 million yen,” “4–4.99 million yen,” “5–5.99 million yen,” “6–6.99 million yen,” “7–7.99 million yen,” “8–8.99 million yen,” “9–9.99 million yen,” “10–14.99 million yen,” and “15 million yen or more”. Columns 3–8 show ordinary correlation coefficients. Columns 9–14 show partial correlation coefficients controlled for sex, age, BMI, GMV, income, education, tenure, and manager.

Abbreviations: Age = years old, BMI = body mass index (kg/m^2^) computed from height and weight, CCA = corpus callosum, CCI = cingulum cingulate, Education = years of schooling, GMV = gray matter volume, Manager = manager 1 and otherwise 0, Sex = male 1 and female 2, SHS = subjective happiness scale, SLF = superior longitudinal fasciculus, Tenure = number of years working in current job, UFA = uncinate fasciculus, WRP = work role performance, WRP‐T = total score of work role performance.

* *p* < 0.05.

** *p* < 0.01.

*** *p* < 0.001.

**FIGURE 4:**
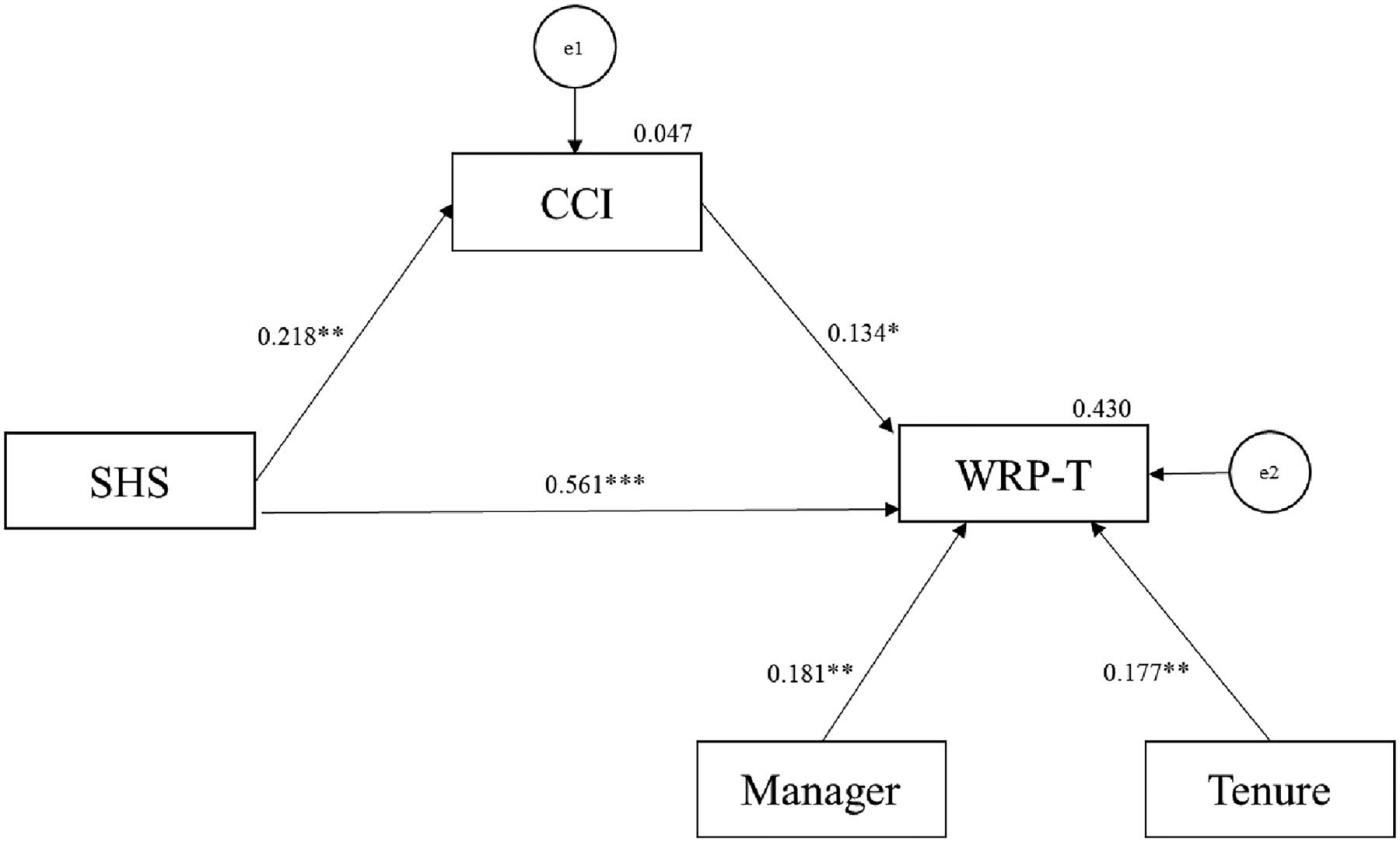
Path analysis results. The figures are standardized (*β*). Sex, age, BMI, GMV, income, and education were not associated with any of the variables and were therefore excluded from the model. *n* = 137; *p < 0.05; **p < 0.01; ***p < 0.001. CCI = cingulum cingulate, e = error term, Manager = manager 1 and otherwise 0, SHS = subjective happiness scale, Tenure = number of years working in current job, WRP‐T = total score of work role performance.

Mediation tests were then conducted using 5000 bootstrap samples, maximum likelihood estimators, and 95% bias‐corrected confidence intervals, as shown in Figure 4. The assessment of the model fit followed Kline (2023), who advocates the use of chi‐square tests, comparative fit index (CFI), root mean square error of approximation (RMSEA), and standardized root mean square residual (SRMR). The acceptable ranges of values for the indices are as follows: chi‐square values from 0 to 3 (McIver and Carmines 1981); CFI ≥ 0.90 (Hu and Bentler 1999); RMSEA < 0.06 (Hu and Bentler 1999); SRMR < 0.08 (Hu and Bentler 1999). Overall, the mediation model explained the data well (*χ* ^2^ = 2.826, df = 5, *χ* ^2^/df = 0.565, *p* = 0.727, CFI = 1.000, RMSEA = 0.000, and SRMR = 0.041). Next, we evaluated the mediating role of apathy on the relationship between SHS and WRP‐T. The results showed that the direct effect of SHS on WRP‐T was positive and significant (*b* = 1.445, *p* = 0.000) and that the indirect effect was also positive and significant (*b* = 0.075, *p* = 0.027). Thus, CCI was shown to partially mediate the relationship between SHS and WRP‐T. An overview of the mediation analysis is presented in Table 2.

**TABLE 2:** Summary of mediation analysis.

Relationship	Direct				Indirect				Conclusion
	Effect	Confidence interval		*p* value	Effect	Confidence interval		*p* value	
		Lower	Upper			Lower	Upper		
		Bound	Bound			Bound	Bound		
SHS → CCI → WRP‐T	1.445	1.099	1.795	0.000	0.075	0.005	0.222	0.027	Partial mediation

*Note*: The figures are unstandardized (*b*).

Abbreviations: CCI = cingulum cingulate, SHS = subjective happiness scale, WRP‐T = total score of work role performance.

## 5 Discussion

In this study, analysis of healthy men and women showed that FA of CCI and SLF were significantly positively correlated with WRP. Among them, CCI showed a broad correlation with three performances (proficient, adaptive, and proactive) corresponding to three roles (individual, team, and organizational). On the other hand, SLF showed a correlation only with adaptive performance of three roles (individual, team, and organizational). These relationships did not change even when partial correlations were controlled for sex, age, BMI, GMV, income, education, tenure, and manager.

Previous studies have revealed a relationship between FA of the CCI and SLF and apathy in AD patients (Hahn et al. 2013; Kim et al. 2011; Smith et al. 2006; Starkstein et al. 1993). Apathy is defined as a quantitative decrease in spontaneous or goal‐directed behavior and often makes it difficult for patients to retrieve information from memory, generate new rules or strategies, or change mental and behavioral sets (Levy 2012). FA can also be altered by lifestyle factors in healthy individuals without specific disorders (Jamieson et al. 2021; Kokubun, Nemoto, and Yamakawa 2024; Pineda et al. 2021; Xu et al. 2023). Therefore, the results of this study that deficits of CCI and SLF, which is related to suppression of apathy, make it difficult for healthy individuals to perform the work by proficient, adaptive, and proactive way in the workplace at the individual, team, and organizational levels is plausible.

The CCI is a WM fiber tract underlying the cingulate gyrus, which is the only communication pathway between the cingulate cortex and other brain regions, such as the prefrontal cortex, parietal lobe, temporal cortex, and thalamus (Domesick 1970). The cingulate gyrus is a relatively recent structure formed during human evolution (Allman et al. 2001) and is involved in emotion, action, memory (Bubb, Metzler‐Baddeley, and Aggleton 2018; Rolls 2019), attention processes (Cohen et al. 1999), and performance monitoring (Lavin et al. 2013; Van Noordt and Segalowitz 2012) in conjunction with the limbic systems. Previously, functional MRI (fMRI) studies confirmed that the cingulate gyrus was significantly activated, whereas subjects with schizophrenia were engaged in a task (Salgado‐Pineda et al. 2004). Consistent with this, studies in healthy subjects also showed that FA in the CCI was related to cognitive functions, such as sustained attention and working memory (Takahashi et al. 2010).

It has also been shown that the CCI correlates with default mode functional connectivity (Bubb, Metzler‐Baddeley, and Aggleton 2018; Van Den Heuvel et al. 2008). Activation of the CCI has been found to be involved in theory of mind (ToM) and mentalizing (Frith and Frith 2006; Mitchell 2009; Molenberghs et al. 2016). Furthermore, recent studies have shown that high FA in the CCI is related to positive emotional empathy (Ziaei et al. 2021). Meanwhile, reduced WM integrity in the CCI is one of the brain features of autistic patients (Travers et al. 2012; Ameis and Catani 2015) and has been shown to be closely related to impairments in ToM, emotion recognition, empathy, social communication, and other social functions, as well as depression (Barch et al. 2022; Li et al. 2017). Therefore, the result of this study that CCI is positively correlated with the broad facets of WRP is consistent with a series of previous studies.

On the other hand, the SLF connects the frontal and parietal lobes and is related to social information processing (Barbey, Colom, and Grafman 2014) and is composed of three regions: SLF‐1, which is responsible for spatio‐motor functions including saccades, voluntary attention, mental imagery, and motor sequences; SLF‐3, which is responsible for non‐spatio‐motor functions including working memory, mirror neurons, semantic and phonological processing, numerical manipulation, response inhibition, automatic attention capture, decision‐making, and emotion processing; and SLF‐2, which is closely related to both clusters and is involved in all of the above functions (Parlatini et al. 2017; Taçyıldız et al. 2024). Previous studies have shown a correlation between the integrity of the SLF and visuospatial cognitive functions (Koshiyama et al. 2020; Tamnes et al. 2010; Urger et al. 2015), working memory performance (Koshiyama et al. 2020; Tamnes et al. 2010; Vestergaard et al. 2011), attention processing (Chica et al. 2018), sustained attention (Klarborg et al. 2013), fine motor skills such as manual dexterity (Hyde et al. 2021), imitation, and social communication (Fishman et al. 2015; Lo et al. 2017). Similarly, it is not surprising that people with abnormalities in the WM microstructure of the SLF and low efficiency in visuospatial cognition, working memory, attention processing, motor control, and imitation feel unable to adapt well to changes in the workplace because they cannot adequately recognize information from the environment, input and output the recognized information, or convert the input and output information into movement or communication. The results of this study showing a positive correlation between SLF and adaptive performance are consistent with a series of previous studies.

Furthermore, this study showed that SHS was broadly correlated with WRP facets as well as CCI, and that SHS may enhance WRP‐T. This result is in line with previous studies showing that happiness and well‐being enhance performance (Al‐Hawari, Bani‐Melhem, and Shamsudin 2019; Bani‐Melhem, Zeffane, and Albaity 2018; Khan and Abbas 2022; Pannells and Claxton 2008; Semedo, Coelho, and Ribeiro 2017; Steptoe 2019). In addition, CCI was shown to partially mediate the relationship between SHS and WRP‐T, and WRP‐T is most likely to increase when SHS is accompanied by an improvement in CCI. These results are consistent with studies showing a relationship between brain structures related to the cingulate cortex and happiness, well‐being (Kokubun, Nemoto, and Yamakawa 2022; Maeda et al. 2021; Matsunaga et al. 2016), and apathy (Bruen et al. 2008; Hahn et al. 2013; Kim et al. 2011; Migneco et al. 2001; Smith et al. 2006; Starkstein et al. 1993).

Previous studies have shown that work‐related attitudes such as organizational commitment and work engagement are broadly correlated with the facets of the WRP, like the findings of the CCI and SHS in this study (Dhir and Shukla 2019; Griffin, Neal, and Parker 2007; Fletcher 2016). In addition, like the findings of the SLF in this study, openness to change was positively correlated with adaptive performance in the WRP (Griffin, Neal, and Parker 2007). On the other hand, in the relationship between the WRP and the Big Five model of personality traits, only some of the correlations hypothesized by previous studies were observed, such as the positive correlation between openness to experience and proactive performance (Neal et al. 2012). The results of Neal et al. (2012) are consistent with recent studies using more experimental methods that have found little relationship between the Big Five personality traits and performance on behavioral decision‐making tasks (Buelow and Cayton 2020). In addition, recent meta‐analyses in neuroscience research have revealed that correlations between brain structure and personality are low or poorly reproducible, regardless of whether they are gray or WM (Avinun et al. 2020; Chen and Canli 2022). Thus, the relationship between psychological variables that are not directly related to work and that are distinct from personality traits and WRP was unclear. The current study is the first to show that indicators reflecting healthy individuals’ happiness and brain microstructure, which are related to a variety of nonwork factors, are positively correlated with the diverse roles and performance that characterize modern work.

Apathy can occur not only due to mental illnesses, such as AD, but also due to unfavorable conditions such as workplace mobbing and bullying, accompanied by reduced self‐esteem and reduced concentration during the worktime (Bambi et al. 2019; Colaprico et al. 2023). Consistent with this, CCI and SLF, which were correlated with WRP in this study, have been linked to apathy (Hahn et al. 2013; Kim et al. 2011; Smith et al. 2006; Starkstein et al. 1993) and racial discrimination trauma (Fani et al. 2022) in previous studies. Furthermore, the ACC, which is connected to CCI, is involved in the recognition and evaluation of socioeconomic justice, morality, and fairness, such as whether outcomes match expectations, as well as whether the processes that brought about these outcomes match social or moral norms (Boksem and De Cremer 2010). In addition, the ACC functions as an “emotional mirror neuron” system, allowing individuals to perceive the emotions of others through neurons and signal both direct pain and the observed pain of others (Schneider et al. 2020). Thus, the results of this study suggest that damage to brain microstructures may mediate the phenomenon in which workplace devastation caused by bullying, mobbing, discrimination, injustice, and so forth leads to reduced work performance accompanied by reduced well‐being not only in direct victims but also in surrounding observers. This means that the quality of human resource management in an organization has a more serious and lasting impact on people and organizations than previously believed, and that managers and administrators must work more seriously to improve justice, morality, and fairness, which is the basis of employee well‐being and productivity in the workplace (Colquitt and Zipay 2015; Eib, Leineweber, and Bernhard‐Oettel 2022).

## 6 Conclusion

We analyzed the relationship between SHS, FA, and WRP using data from 137 healthy men and women recruited in Japan. The results showed that CCI and SLF FA were positively correlated with WRP and that CCI partially mediated the relationship between SHS and WRP. This study is the first to show that indicators reflecting healthy individuals’ happiness and brain microstructure, which are related to a variety of nonwork factors, are positively correlated with the diverse roles and performance that characterize modern work.

## Author Contributions

**Keisuke Kokubun**: writing–original draft, formal analysis, conceptualization, methodology, software. **Kiyotaka Nemoto**: conceptualization, software, validation, resources, writing–review and editing. **Yoshinori Yamakawa**: data curation, supervision, project administration, writing–review and editing, funding acquisition.

## Ethics Statement

This study was approved by the Ethics Committee of Kyoto University (Approval Number 27‐P‐13) and Tokyo Institute of Technology (Approval Number A16038) and was conducted following the institute's guidelines and regulations.

## Consent

All participants provided written informed consent before participation, and their anonymity was maintained.

## Consent for Publication

All participants gave consent for the publication of the results of this study.

## Conflicts of Interest

The authors declare no conflicts of interest.

### Peer Review

The peer review history for this article is available at https://publons.com/publon/10.1002/brb3.70334.

## Data Availability

The datasets generated during the current study are not publicly available but are available from the corresponding author upon reasonable request.
